# Bioactive glass-based fibrous wound dressings

**DOI:** 10.1093/burnst/tkac038

**Published:** 2022-09-28

**Authors:** Shahin Homaeigohar, Meng Li, Aldo R Boccaccini

**Affiliations:** School of Science and Engineering, University of Dundee, Dundee DD1 4HN, United Kingdom; Institute of Biomaterials, Department of Materials Science and Engineering, University of Erlangen-Nuremberg, 91058 Erlangen, Germany; Institute of Biomaterials, Department of Materials Science and Engineering, University of Erlangen-Nuremberg, 91058 Erlangen, Germany

**Keywords:** Scaffolds, Fibers, Angiogenesis, Wound healing, Bioactive glass

## Abstract

Since the discovery of silicate bioactive glass (BG) by Larry Hench in 1969, different classes of BGs have been researched over decades mainly for bone regeneration. More recently, validating the beneficial influence of BGs with tailored compositions on angiogenesis, immunogenicity and bacterial infection, the applicability of BGs has been extended to soft tissue repair and wound healing. Particularly, fibrous wound dressings comprising BG particle reinforced polymer nanofibers and cotton-candy-like BG fibers have been proven to be successful for wound healing applications. Such fibrous dressing materials imitate the physical structure of skin’s extracellular matrix and release biologically active ions e.g. regenerative, pro-angiogenic and antibacterial ions, e.g. borate, copper, zinc, etc., that can provoke cellular activities to regenerate the lost skin tissue and to induce new vessels formation, while keeping an anti-infection environment. In the current review, we discuss different BG fibrous materials meant for wound healing applications and cover the relevant literature in the past decade. The production methods for BG-containing fibers are explained and as fibrous wound dressing materials, their wound healing and bactericidal mechanisms, depending on the ions they release, are discussed. The present gaps in this research area are highlighted and new strategies to address them are suggested.

Highlights

- Wound healing bioactive glass (BG) nano/microfibers are comprehensively reviewed.
- BG fibrous wound dressings are made from BG particle reinforced polymer fibers and cotton-candy-like fibers.
- Fiber drawing, electrospinning and laser spinning are typical fabrication techniques for BG fibers.
- BG fibrous dressings release biologically active ions for angiogenesis and wound healing.
- The positive impact of BG fibers on wound healing can be related to their immunogenic impact.

## Background

Across the world, skin disruptions and wounds in chronic and even acute form endanger patients’ welfare and indirectly challenge the healthcare systems. For instance, according to the statistics published in 2015 [1], management of chronic wounds costs the National Health Service (NHS) of the UK between £4.5 and £5.1 billion per year. In the UK, 200,000 patients suffer from a chronic wound [2], caused by increasing the incidence of diabetes and obesity and by an aging population. Approximately 1–2% of the general population will experience a chronic wound and up to 25% of diabetic patients will develop an ulcer [3]. To address this crisis, the global advanced wound care market including wound dressings is expected to reach £18.6 billion by 2024 from £14.8 billion in 2019 [4]. Such a market has been growing not only in terms of customer numbers but also technology-wise. To address the dynamic nature of wound healing and its multidimensional objectives, the wound care market is transitioning from classic protective barriers into advanced, bioactive wound dressings, interacting with the wound by stimulating and managing cell migration and the sequence of healing events including inflammation, proliferation and remodeling [5].

The inflammatory phase starts upon fulfillment of hemostasis and whereby pathogens are eliminated from the wound bed. To accomplish this objective, vascular permeability is enhanced via vasodilation, allowing for accumulation of monocytes and neutrophils inside the wound milieu [5]. The proliferation phase follows inflammation after nearly 3 days and proceeds with the formation of collagen and ground substance, driven via the activity of fibroblasts. The fibroblasts existing in the wound bed and those originating from blood, proliferate and migrate, whereby forming wound granulation tissue alongside a new extracellular matrix (ECM). Moreover, some fibroblasts are differentiated into myofibroblasts to engender wound closure [6]. Over the course of the proliferation step, endothelial cells promptly grow and trigger vascularization within the granulation tissue. Eventually, the remodeling (maturation) of the wound tissue results in formation of normal tissue after 2–3 weeks [7].

Compared to the classic dressings made as foams, films, hydrogels and sponges, fibrous wound dressings are an emerging class with distinct advantages. Fibrous dressings provide notable structural resemblance with the ECM in terms of porosity, morphology and mechanical properties [8–10]. Apart from structural similarity, fibrous systems can be made of inorganic and organic materials, providing the necessary biochemical cues for the cells involved in the wound healing process. Among the proposed fibrous materials for wound healing applications, bioactive glass (BG), either as a filler or as the main fiber material, has proven to be a promising candidate with distinct advantages for wound healing, angiogenesis and antibacterial activity through the release of supportive biologically active ions.

BGs are highly bioactive inorganic materials with different compositions, which can be shaped into different physical forms (particulate and fibrous), allowing their implementation as a novel class of wound dressing materials [10–12]. BGs are primarily renowned for their well-investigated potential for bone repair [13,14]. Additionally, in recent years, they have been considered for soft tissue repair [11]. In this regard, the original silicate BG developed by Hench, i.e. 45S5 bioactive glass [15], has been extensively investigated and proposed for various clinical applications. For instance, a fibrous structure made of 45S5 BG has been employed for the treatment of soft tissue ulceration and skin repair [10]. This BG can offer a controlled ion release and ion exchange process and induces formation of a hydroxyapatite (HA) layer upon immersion in the body fluid [10]. As a result, better healing conditions are realized for a soft tissue such as skin, via activation and upregulation of healing factors, including antigen hematopoietic form precursor (CD44), fibroblast growth factor receptor precursor (N-sam), vascular cell adhesion protein precursor, vascular endothelial growth factor (VEGF) precursor, and fibronectin receptor beta subunit [16]. The main cells involved in wound healing are thus provoked to further proliferate and grow when subjected to such factors and accumulate in areas adjacent to the BG surface, thereby forming new skin tissue [10].

In the current review, we aim to highlight the emerging role and significance of BG fibrous materials for wound healing. The involved healing mechanisms are discussed and different types of BGs in terms of composition and form will be introduced, while mentioning the pros and cons of each type. We intend to unravel the available gaps in this research area and propose new solutions to the currently available shortcomings. This might prompt researchers to try new perspectives and approaches. We also discuss commercial BG fibrous dressings and highlight their healing features. It is worthy to note that this topic has been insufficiently studied as reflected in the number of publications coming up in ‘Web of Science’, totaling 26 (bioactive glass fiber) and 18 (bioactive glass + electrospinning + wound healing). This review is thus of relevance to those interested in exploring applications of fibrous BGs in wound healing.

## Review

### Different types of BGs

BG is a bioactive material that upon *in vivo* implantation develops a HA surface layer, thereby creating a robust interface with hard tissues (e.g. bone and tooth). Such a HA layer can be also of relevance to enable bonding with soft tissues (e.g. skin) [17]. The chemical composition of BGs can be tailored by inclusion of biologically active ions that provoke particular cellular activities [18,19]. In general, depending on the glass network former, BGs can be classified as silicate BGs, borate BGs and phosphate BGs [19].

#### Silicate BGs

Silicate BGs were for the first time developed around 50 years ago in the seminal work of Hench and co-workers [20]. Thereafter, the 45S5 BG composition (45 wt.% SiO_2_, 24.5 wt.% CaO, 24.5 wt.% Na_2_O, and 6.0 wt.% P_2_O_5_) has been extensively investigated for various biomedical applications [21]. From a structural standpoint, this silicate BG comprises a 3D glass-forming SiO_2_ network [11]. The main compositional characteristics that synergistically bring about bioactivity of 45S5 BG include: (1) inferior SiO_2_ amount as compared to chemically resistant silicate glasses (e.g. soda-lime glass), (2) relatively high content of CaO and Na_2_O (glass network modifiers), and (3) high CaO/P_2_O_5_ ratio [11]. An alternative silicate BG is the composition 13–93 BG (53% SiO_2_, 20% CaO, 6% Na_2_O, 4% P_2_O_5_, 12% K_2_O, 5% MgO in wt.%) [22], which has been developed to exhibit less bioactivity than 45S5 BG and to facilitate fiber fabrication from molten glass [23–25].

#### Borate BGs

Particular glass-forming systems like borate glasses of certain compositions have also been shown to be bioactive [10]. Borate BG, particularly 13-93B3 glass (54.6% B_2_O_3_, 6% Na_2_O, 22.1% CaO, 7.9% K_2_O, 1.7% P_2_O_5,_ 7.7% MgO in mol.%) [26], is more biodegradable and bioactive compared to silicate BGs, thus offering distinct potential for bone and soft tissue repair [27–29]. Borate BGs, thanks to a lower chemical resistance than silicate 45S5 and 1393 BGs, degrade in an even shorter time than silicate BGs and transform majorly to an HA-based material [11,27–29]. For instance, compared to 45S5 BG, 13-93B3 borate glasses have been reported to react with simulated body fluid (SBF) in a five times shorter time [27]. Borate BGs transform to HA in a similar manner to 45S5 silicate BG, yet without formation of the SiO_2_-rich surface layer [27]. Borate BGs offer promising biological properties, thereby enhancing cell proliferation and cell differentiation *in vitro* [30,31], and promoting tissue ingrowth *in vivo* [32]. Their further application as a drug delivery substrate to address the bone infection problem has also been validated [33–35]. Moreover, borate BGs were rapidly considered for wound healing applications [10]. In this regard, cotton-candy-like fibers composed of 13-93B3 glasses have been proven to be effective in healing diabetic ulcers, most likely due to the release of B and Ca ions that can drive the migration process of epidermal cells and govern the wound healing cascade [36,37].

Despite all the therapeutic pros mentioned earlier, borate BGs might induce adverse biological responses, due to the potential toxicity of the released borate ions, (BO_3_)^3−^, at relatively high concentrations [11]. As reported by Brown et al. [38], some borate BGs trigger cellular toxicity when tested under ‘static’ *in vitro* conditions, while remain non-harmful to cells under ‘dynamic’ testing conditions. Substituting silica in 13-93 BG with B_2_O_3_ has led to a very popular borate glass, designated as 13-93B3. This glass has been shown to be toxic *in vitro* against murine MLO-A5 osteogenic cells [32]. In contrast, such a composition has not shown any toxic effects *in vivo*; on the contrary, the glass encouraged tissue regeneration and ingrowth in rat models [39].

One important characteristic of borate BGs is the tailorability of their degradation rate through partial or total replacement of SiO_2_ with B_2_O_3_ in silicate 45S5 or 13-93 BGs, for instance, to achieve a borosilicate or borate BG [27,28,40]. Moreover, the addition of biologically active ions to a basic borate glass composition provides a useful approach to enhance the biological activity of the BG. A typical example is the addition of Cu ions to 13-93B3 to induce an angiogenic effect [41].

#### Phosphate BGs

Other than silicate- and borate-based BGs, phosphate BGs, comprising a P_2_O_5_ glass-forming network alongside Na_2_O and CaO modifiers, have also been synthesized for biomedical applications [42,43]. Similar to borate BGs, the degradation rate of phosphate glasses (thus their interaction with cells) can be modulated by tailoring the glass’s chemistry (composition). Such a characteristic further expands the clinical potential of phosphate BGs [11]. The cellular response to phosphate BGs has been tuned via stabilization of the glass network and by controlling their degradation rate, which can be achieved by inclusion of various oxides such as B_2_O_3_, TiO_2_, MgO, ZnO and CuO [44–48]. Phosphate BGs can be formed as microfibers, which enables their applicability in wound healing [49]. For instance, recently, gallium and cerium ion-doped phosphate BG fibers (18MgO–10CaO–24Na_2_O–45P_2_O_5_–3Ga_2_O_3_/CeO_2_ mol.%) have been investigated for wound dressing application [49].

### BG-induced wound healing mechanisms

The volume of research on osteogenesis induced by BGs notably prevails over the number of studies dealing with the potentials of BGs for soft tissue regeneration and wound healing. However, the encouraging effect of BGs on angiogenesis has been already validated [50,51]. Given the fact that over the course of the granulation phase, angiogenic rooting of new vessels is of high importance for delivering nutrients and oxygen to the cells present in the wound bed to induce wound healing [52], BG-based therapies that promote angiogenesis can be particularly effective [53]. There are diverse strategies that potentially support angiogenesis, for instance, by biohybrid constructs that contain pro-angiogenic factors such as platelet-derived growth factor (PDGF), VEGF, and basic fibroblast growth factor (bFGF) [54–56]. Yet, this concept is typically costly, might impose undesired biological consequences particularly when it involves supra-physiological doses [57,58], and declines bioactivity [59,60]. In contrast, inorganic pro-angiogenic factors (ions) appear to offer advantages such as optimum stability, low cost and higher clinical safety as compared to the mentioned growth factors [61,62]. For instance, Cu ions have been shown to play a crucial role in the angiogenic response [63] through control of the expression of hypoxia-inducible factor (HIF-1α), thereby simulating hypoxia, that notably contributes to formation of blood vessels [62]. Cu^2+^ ions provoke the proliferation of endothelial cells and thus angiogenesis through mediation of the release of cytokines and VEGF [64–66]. Additionally, the release of such ions can upregulate growth factor-β (TGF-β), as a pro-angiogenic factor, in diabetic wounds [67,68], which reduces the risk of ischemia in skin flaps [69]. The presence of borate ions in human keratinocyte cultures, even in millimolar concentration, can upregulate matrix metalloproteinases MMP-2 and MMP-9, thereby driving the migration of these cells and promoting remodeling of granulation tissue [70]. As a result, development of BG systems releasing such pro-angiogenic ions can be regarded as a promising, simple strategy for wound healing. In this regard, Jung synthesized BG fibrous scaffolds made of 45S5 and 13-93B3 (with and without CuO), that could release pro-angiogenic ions and encourage soft tissue ingrowth *in vivo* [71]. Based on a histological analysis, it was shown that the soft tissue growing into the Cu-doped 13-93B3 fibrous scaffold contained a larger microvascular density compared to that found in the 45S5 fibrous scaffold [71].

As reported by Wray in 2011 [72], two kinds of borate BG microfiber dressings composed of 13-93B3 and 13-93B3 with 0.4 wt.% CuO were applied for the purpose of healing cutaneous wounds clinically. According to this study, both BG microfiber dressings were able to optimally heal the wounds in 66% of the patients who took part in the study [72]. Interestingly, 13-93B3 dressing promoted re-epithelialization, while the Cu-doped 13-93B3 BG dressing provoked the formation of granulation tissue. As a result, collectively BG microfibers caused tissue regeneration with proper vascularization [72].

**Figure 1.**
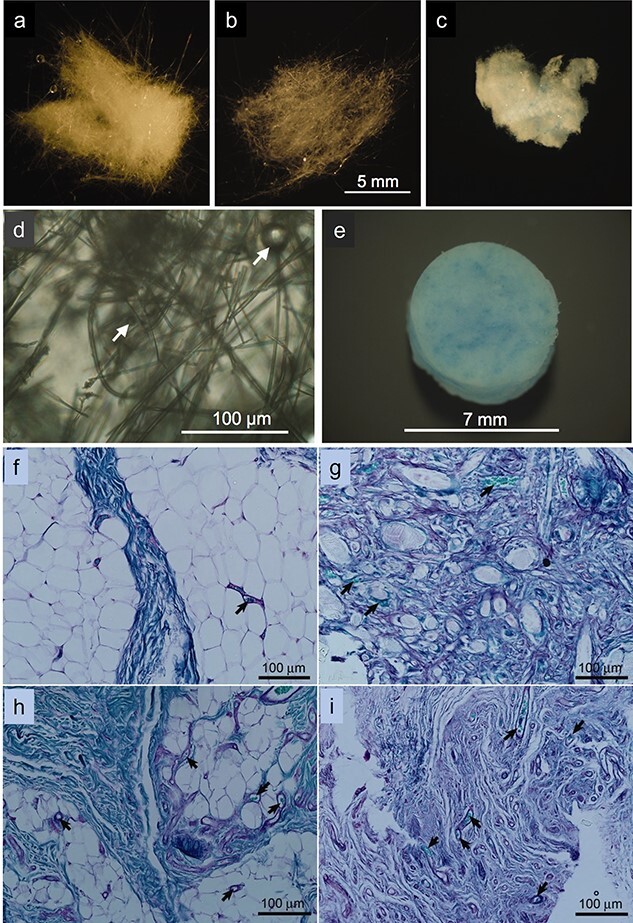
Cotton-like microfibers composed of: **(a)** 45S5, **(b)** 13-93B3, and **(c)** Cu-doped 13-93B3 BGs. **(d)** Image of 13-93B3 BG microfibers at a high magnification (the arrows mark glass beads). **(e)** Camera image of the Cu-doped 13-93B3 BG microfiber implant. The periodic acid Schiff (PAS)-stained sections of soft tissue exposed (for 4 weeks) to implanted BG microfibers composed of: **(f)** none (sham control); **(g)** 45S5, **(h)** 13-93B3, and **(i)** Cu-doped 13-93B3 (the arrows mark the microvessels found in the tissues). Reproduced with permission from [36]. Copyright 2014, John Wiley and Sons

Lin et al. [36] quantitatively characterized the angiogenic response of a soft tissue wound to borate BG microfibers (Figures 1b, c, representing the neat and Cu-doped 13-93B3 microfibers, respectively) in comparison to 45S5 silicate BG microfibers (Figure 1a) and sham implant controls. Figures 1d, e show a high magnification image and a camera image of the borate BG microfibers implanted in an animal model, respectively. As shown in Figures 1f–i, 13-93B3 BG microfibers doped with copper (0.4 wt.%) raised angiogenesis more notably than 45S5 BG microfibers and sham controls did. The superior angiogenic behavior of such BG microfibers stems from beneficial biological effects of the released copper and borate ions on endothelial cell proliferation and vessel formation, as mentioned earlier. In general, the ions released from BG microfibers including silica and copper ions might also induce collagen synthesis, thereby forming a fibrous tissue in addition to that resulting from the inflammatory response against the implanted microfibers [64]. Additionally, possible cytotoxicity of the released borate ions over a long time (4 weeks) was investigated via a histological analysis on a kidney tissue obtained from rats after subcutaneous implantation of the borate BG microfibers. This study did not show any sign of long-term histopathological consequences in kidney [36].

The positive impact of BGs on wound healing can be also related to their promising immunogenic impact. BGs can alter the local microenvironment, thereby modulating the activities of macrophages via physicochemical and biological cues [73,74]. Dong et al. [74] have indicated that the ions released from 45S5 BG particles can not only polarize macrophages as M2 phenotype but also drive them to largely produce anti-inflammatory growth factors. These chemokines and cytokines, such as VEGF, bFGF and TGF-β, are known to play several important roles in the tissue regeneration cycle [75,76]. Among the mentioned growth factors, TGF-β and VEGF, have been shown to employ and attract repairing cells such as fibroblasts and endothelial cells [77], thereby promoting collagen deposition, re-epithelization and vascularization. TGF-β is a cytokine that is involved in all steps of the wound healing process [78]. It provokes the proliferation of fibroblasts and thus the synthesis of ECM. As a result, provisional granulation tissue forms within the wound milieu [79]. Conversely, VEGF and bFGF can support angiogenesis and vascularization during the wound healing process [80]. VEGF as a pro-angiogenic growth factor provokes the migration of endothelial cells and helps them assemble as capillaries [81]. Moreover, bFGF increases the population of new capillaries in the wound bed [82].

### Production methods of BG-based fibers

The production method of BG fibers totally depends on the glass processability and melting behavior. For instance, given the narrow sintering window of 45S5 BG, fiber production through typical melt-spinning methods is quite challenging and results in glass crystallization [83]. In contrast, the composition of 13-93BG enables fiber drawing from its melt, though in micron size [84]. As mentioned earlier, nanofibers are more in demand, considering their biomimicry effect and ability to simulate the collagenous ECM in terms of morphology and topography [85]. Therefore, various BG nanofiber production techniques have been developed that suit rheological properties and crystallinity of the BG composition. In this regard, laser spinning and electrospinning are well-known strategies that have advanced the development of BG nanofibers of high quality (desired composition) and quantity.

#### Laser spinning

**Figure 2.**
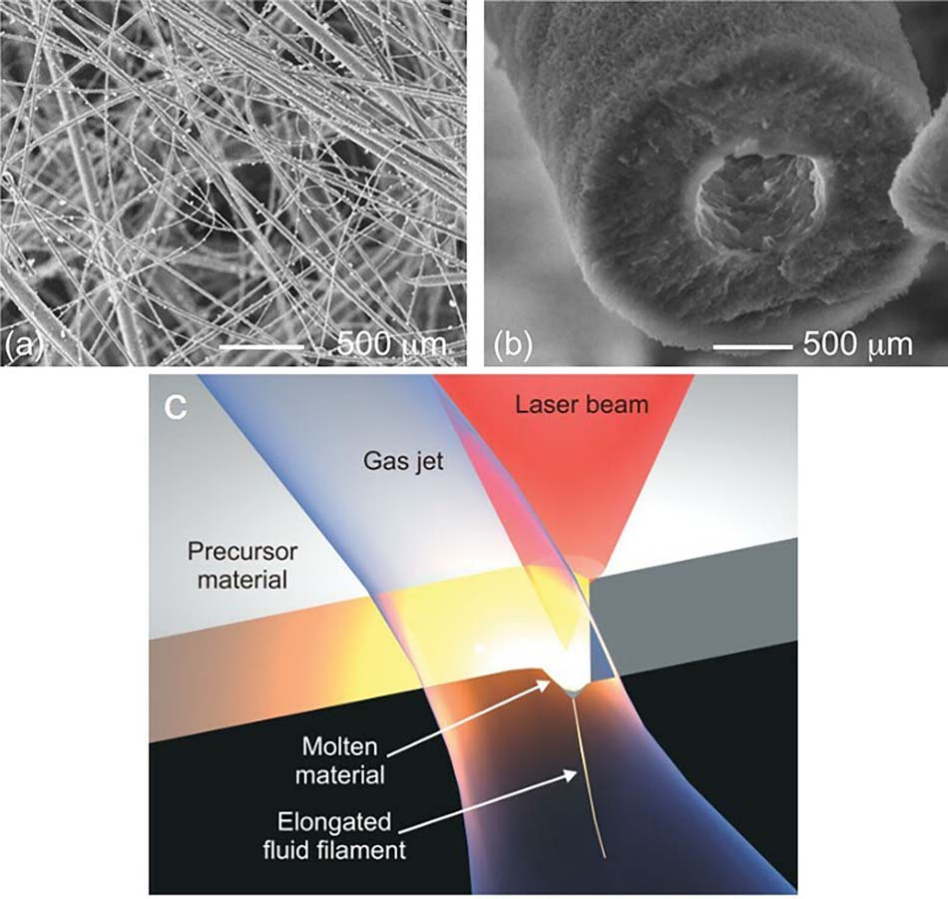
Scanning electron microscopy images showing: **(a)** 45S5 BG nanofibers produced through the laser spinning process, **(b)** conversion of a 45S5 BG nanofiber to a hydroxycarbonate apatite tube after immersion in simulated body fluid for 48 h. **(c)** The laser spinning method that employs a powerful laser to get a small fraction of a precursor material melted. Meanwhile, a high-velocity gas jet draws and cools the molten material, forming a nanofiber. Reproduced with permission [88]. Copyright 2009, John Wiley and Sons

Laser spinning allows for production of BG micro/nanofibers in a large quantity [86]. The technique is applicable for any specific, predefined chemical composition of BGs with no need for inclusion of chemical additives or a subsequent thermal treatment [87]. For instance, with respect to 45S5 BG, the only possible nanofiber fabrication technique that can produce amorphous 45S5 BG nanofibers is laser spinning [83], as shown in Figure 2a. In this method, nanofibers are made via laser irradiation on a 45S5 BG monolith, resulting in formation of a small bath of molten glass, that is later spun (stretched and cooled) by a gas jet emanating from a supersonic nozzle [88]. As a result of fast cooling that hinders crystallization, an amorphous BG non-woven nanofiber is created that comprises nanofibers as small as 200 nm to 300 nm in diameter. The laser spinning process is highly time-efficient and BG nanofibers can be produced in a few microseconds. In addition to 45S5 BG, Quintero et al. [88] succeeded in producing 52S4.6 silicate BG nanofibers (52.27 SiO_2_, 0.45 Al_2_O_3_, 24.71 CaO, 2.30 P_2_O_5_, 20 Na_2_O, 0.21 K_2_O, 0.01 Fe_2_O_3_, 0.03 TiO_2_ mol.%), as well. Interestingly, the nanofibers could be promptly transformed to hydroxycarbonate apatite (HCA) tubes (Figure 2b) upon immersion in SBF, thanks to their nanoscale diameter (size) and composition [88].

Figure 2c schematically illustrates the laser spinning process, in which a pendant drop of the molten glass is exposed to a high-velocity gas jet that forcefully stretches and cools the melt [88]. The cooling speed is extremely high and as a result the spun nanofibers are amorphous. The superfast nature of the process involving high elongation forces enables production of nanofibers with extraordinary length/diameter ratios of, e.g. 1,000,000 : 1 in less than one second [88]. The relative movement of the laser beam against the plate of the precursor material, i.e. 45S5 and 52S4.6 BG plates, creates a cut that persistently supplies the melt and thus allows for production of dense BG nanofiber mats in minutes [88].

#### Electrospinning

**Figure 3.**
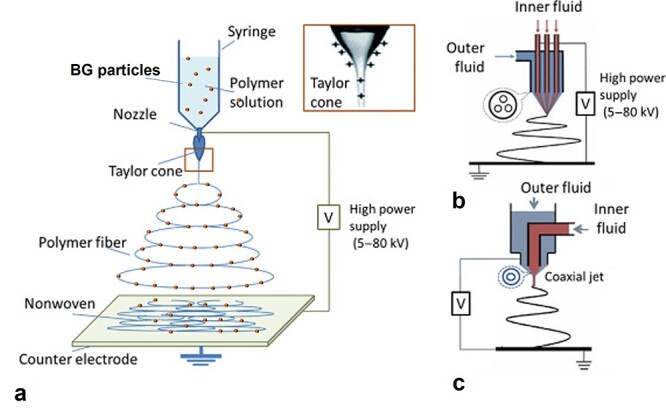
Schematic illustration of electrospinning of a BG particle/polymer suspension in various set-ups with different nozzle configurations: **(a)** single nozzle, **(b)** multichannel nozzle, and **(c)** coaxial nozzle. Reproduced and re-drawn (section a) with permission [94]. Copyright 2019, Elsevier

Electrospinning enables production of polymeric submicron fiber mats featuring an extraordinary surface area that can be engineered in terms of chemistry and topography, adjustable porosity, and conformability over an extensive range of objects with different shapes and sizes [8,9,89–92]. The technique can be upgraded to produce BG-polymer composite fibers that are characterized with the presence of BG surface nanodomains along the polymer fiber matrix. In this simple approach, as shown in Figures 3a–c, a polymer solution containing BG particles is constantly (with a fixed feed rate) infused through a nozzle electrified by a high DC voltage supplier and is converted to a polymer jet flying toward a grounded collector. The structural properties and morphology of electrospun nanofibers including diameter, surface porosity (roughness), and alignment are predetermined by the device operating parameters, polymer solution properties, and nearby environment conditions [93]. Moreover, multiphasic nanofibers can be produced by electrospinning set-ups containing multichannel and coaxial nozzles (Figures 3b, c, respectively) [94].

Electrospinning allows for simple production of nanofibers made of natural and synthetic polymers individually or blended with other polymers. Additionally, depending on the target application and desired structural and biological properties, inorganic–organic composite nanofibers can be developed. In this regard, BG-(bio)polymer suspensions have been electrospun to provide composite nanofiber systems benefitting from a brittle yet bioactive inorganic BG phase alongside the bioinert yet flexible organic (bio)polymer phase [95]. For instance, BG–PCL (polycaprolactone) composite nanofibers have been shown to offer proper bioactivity and to stimulate the secretion of alkaline phosphatase by MC3T3 pre-osteoblast cells adjacent to the nanofibers [96,97]. Considering the different chemical nature of the inorganic filler (BG) and the organic (polymer) matrix, interfacial bond strength can be insignificant, leading to a poor distribution of the BG phase and low mechanical properties. One promising solution in this respect is the use of a coupling agent. For instance, 3-glycidoxypropyltrimethoxysilane has been employed to induce formation of a covalent bond between BG particles and gelatine, thereby creating flexible, yet mechanically robust BG-gelatine composite fibers [98]. A large variety of BG-containing biopolymer electrospun fibers has been developed for wound healing application [99–103].

BG nano/microfibers can also be synthesized through a combination of sol–gel process and electrospinning [104]. Such nanofibers are electrospun in a similar manner as polymer nanofibers, though a polymer is added to the inorganic sol. The first electrospun silicate BG nanofibers (as small as 84 nm in diameter) were made of 70 mol.% SiO_2_, 25 mol.% CaO, and 5 mol.% P_2_O_5_ through a combination process comprising sol–gel and electrospinning [83,105]. To enable electrospinning, viscosity of the sol was tailored by addition of polyvinyl butyral/ethanol solution in a small amount. The 70S30C BG nanofibers were also electrospun with inclusion of polyvinyl alcohol in the sol [105]. More sophisticated versions of BG nanofibers such as those made as hollow mesoporous fibers (∼600 nm in diameter) have also been synthesized using high molecular weight poly(ethylene oxide) (PEO) as the phase separation (and then sacrificial) agent [106]. Recently, cotton-like Cu ion-doped BG nanofibers have been developed by a similar sol electrospinning technique [107].

### BG-based fibers for wound healing

#### BG nanoparticle reinforced polymeric nanofibers

Despite all the merits that BGs show for bone regeneration and wound healing such as formation of a calcium phosphate layer in exposure to physiological liquids, enhancing osteointegration, and release of therapeutic ions stimulating different cellular pathways, their application is restricted by their insufficient mechanical properties. As a solution for this shortcoming, BGs have been combined with biodegradable synthetic or natural polymers to create malleable yet bioactive composite nanofibers that can potentially be applied as a wound dressing material.

#### BG/natural (and blend) polymer nanofibers

**Figure 4.**
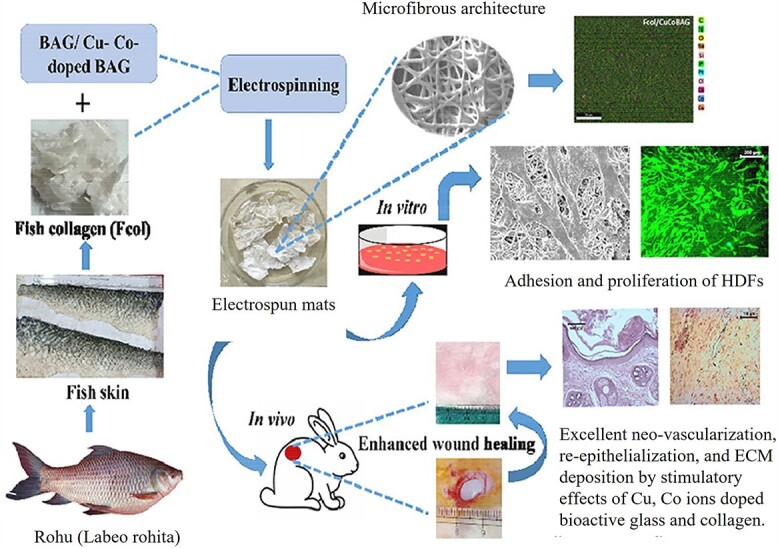
Schematic illustration of the preparation procedure of bioactive glass (BG) (here shown as BAG; Cu- and Co-doped) reinforced fish collagen electrospun fiber mats with improved human dermal fibroblast (HDF) cell response and enhanced *in vivo* wound healing reflected in neovascularization, re-epithelialization, and extracellular matrix (ECM) deposition. Reproduced with permission from [100]. Copyright 2022, ACS

The production of natural polymer nanofibers from biological wastes or bioresources for biomedicine has always been appealing, due not only to their biomimicry but also to their suitable biocompatibility and biodegradability. For instance, fish collagen has been proven to be a biocompatible biomolecule that poorly induces antigenic response and offers promising wound healing effects [108]. However, it is highly costly and thus rarely applicable in biomedicine, unless it is synthesized from economical resources. In this regard, fish collagen can be potentially extracted from biowastes largely produced in fish processing units [109]. The as-prepared collagen *per se* cannot be used as a wound healing material, due to its insufficient thermomechanical properties, leading to fast degradation at the physiological temperature of the human body. One optimum solution for such a bottleneck can be the combination of fish collagen and BG (nano)particles to create a composite system with improved structural and therapeutic properties. In this regard, Zhou et al. [101] developed composite nanofibers composed of BG and Tilapia fish collagen. The as-developed nanofibers were shown to possess improved tensile strength and the ability to inactivate *Staphylococcus aureus* bacteria. Moreover, such nanofibers could raise skin regeneration in the wound bed, indicating their wound healing potential. Very recently, Jana et al. [100] synthesized a microfibrous wound dressing made of fish collagen, derived from *Rohu* (*Labeo rohita*) skin, coupled with a novel formulation of BG doped with Cu and Co. As shown in Figure 4, the structure and composition of the microfibrous dressing were encouraging for human dermal fibroblasts (HDFs) to adhere, spread and proliferate on such a cytocompatible and nontoxic platform. *In vivo* testing with animal models (rabbits) also confirmed an improved wound healing behavior in the presence of the doped BG reinforced fish collagen microfibers. Particularly, enhanced wound closure, homogenously formed epidermis, larger wound maturity, and proper deposition of ECM components including mature elastin and collagen were observed. Additionally, neovascularization was obvious in the wounds treated with the doped BG/fish collagen microfibers, most likely due to the bioactivity of BG and the supportive role of Cu and Co ions along with fish collagen [100]. Eggshell membrane (ESM) is a connective tissue that comprises a thin (60–80 μm) layer of collagen fibers [110]. ESM is in fact a porous biopolymeric fibrous network consisting of protein fibers (80–85%), thereof ∼10% are made of collagen (types I, V and X) [111]. In addition to its fascinating porous structure, ESM provides a proper antibacterial activity that is necessary for wound healing [112]. Employing the interesting characteristics of ESM, Li et al. [113] devised a Cu-doped BG coated ESM for wound healing application. The 5 mol.% Cu-BG/ESM material could provoke angiogenesis by upregulation of VEGF and HIF-1α, in human umbilical vein endothelial cells (HUVECs). Moreover, thanks to the sustained release of Cu^2+^ ions, the system was successful in inactivation of *Escherichia coli* bacteria.

Plant-derived natural polymers, e.g. cellulose, have also been researched to be applied as a carrier for BG and cooperatively for production of composite nanofiber wound dressings. Cellulose is the most abundant natural polysaccharide that can be derived from green resources such as plants, wood, fungi, seaweed and bacteria [114–116]. Thanks to negligible toxicity and carcinogenicity, biocompatibility and biodegradability, cellulose is regarded as a high-potential wound dressing material. Additionally, it can maintain moisture, adequately absorb exudates, expedite granulation and encourage wound healing via fibrogenesis [115,117]. As a derivative of cellulose, methylcellulose (MC) has been electrospun blended with PCL, which assures desirable electrospinning of MC. The MC/PCL blend nanofibers were incorporated with borate BG and Manuka honey to develop a wound dressing material with antibacterial properties [99]. *In vitro* tests based on human keratinocytes (HaCaT cells) and HDFs confirmed that the nanofibrous system can be potentially applied as a wound dressing material [99]. BG nanoparticles have also been employed to reinforce cellulose acetate nanofibers (100–200 nm in diameter) to develop a broad spectrum antibacterial wound dressing material that accelerates wound healing [118]. Another polysaccharide that has been widely studied for biomedical applications is chitosan. Chitosan is also biodegradable, nontoxic, low cost and abundant. Additionally, it accelerates tissue regeneration and induces hemostasis [119]. Moreover, chitosan shows antibacterial activity that is attributed to its capability in binding with sialic acid in phospholipids, thereby challenging the transport of microbiological substances [120]. Such important biological characteristics render chitosan an ideal candidate for fabrication of biomedical systems for wound healing, tissue engineering, drug delivery, among other applications [121]. In a recent study by Sergi et al. [122], chitosan/PEO blend fibers, cross-linked with genipin, were incorporated with several types of BG including 45S5 BG, Sr- and Mg-doped BG and Zn-doped BG. The release of therapeutic ions such as Sr, Mg and Zn ions from chitosan/PEO fibers was reported to potentially raise tissue regeneration. Sr ions can provoke cell proliferation and angiogenesis [50,123], whereas Mg ions can improve migration and proliferation of microvascular cells [124]. Furthermore, Zn ions cause better wound healing and angiogenesis conditions in the wound bed [125,126]. Cerium (Ce)-doped BG particles have also been embedded into chitosan/PEO nanofibers to create antibacterial nanofiber wound dressings with improved mechanical properties, matching those of skin [98]. Ce-doped BGs have been shown to effectively inactivate gram-negative bacteria such as *Escherichia coli*, particularly at Ce concentrations exceeding 5 mol.% [127]. Silk fibers, as the building blocks of the commercial suture Mersilk®, are comprised of a fibroin core encased by sericin, which is an antigenic gum-like protein [128]. Moreover, to confer polymer sutures with bioactivity and antibacterial activity, they have been coated with Ag-doped BG (60% SiO_2_, 2% Ag_2_O, 34% CaO, 4% P_2_O_5_ in mol.%) via a conventional slurry-dipping approach. The as-coated suture could show limited bacterial adhesion, thus a promising antibacterial effect against *Staphylococcus epidermidis* [128].

#### BG/synthetic polymer nanofibers

Synthetic polymers have also been proposed for construction of BG incorporated nano/microfibrous wound dressings. Such a type of polymers is outstanding due to their largely known processing techniques (e.g. electrospinning), desirable physicochemical characteristics and scalability [9]. Such advantages can be crucial for production of wound dressing materials at large scale and in a cost-effective manner. Despite such merits, synthetic polymers are typically bioinert and thus can challenge the removal of wound dressings made thereof after the wound is healed [89]. This shortcoming can be addressed by blending with natural, biodegradable polymers and by incorporation of bioactive (inorganic) materials such as BG. For instance, insufficient number of cell recognition sites on PCL nanofibers and their poor bioactivity are crucial bottlenecks that can be addressed by addition of BG particles. In this regard, silicate (13-93) and borosilicate (13-93BS) BG nanoparticles have been incorporated into poly(glycerol-sabacate)(PGS)/PCL blend nanofibers to create a wound healing material [129]. Thanks to the pro-angiogenetic activity of the ions released from the BG particles, the BG-reinforced PGS/PCL nanofibers can be employed as a wound dressing material. PCL fibers containing pro-angiogenic Co-containing BG particles were also developed to stimulate wound healing without formation of a HCA layer [130]. Co ions were released steadily with a release rate governed by Mg concentration of the BG. As a result of the dissolution of BG, in an *in vitro* study with primary human fibroblasts, HIF-1α was stabilized and VEGF was notably upregulated. Therefore, the composite fibers can potentially activate the HIF pathway, thereby stimulating angiogenesis [130]. In general, as the current literature review indicates, natural polymers seem to be more appealing for the development of BG-incorporated polymer fibers meant for wound healing. Among the few synthetic polymers proposed for such a research objective, undoubtedly, PCL is the most studied polymer. Table 1 tabulates the studies that deal with BG-incorporated PCL nano/microfibers developed for wound healing that have been carried out in the past five years [131–137]. In addition to PCL, bioresorbable poly(glycolide-l-lactide) (PGLA) fibers, constituting Vicryl® (polyglactin 910) surgical sutures, have been coated with Ag-doped BG (60% SiO_2_, 2% Ag_2_O, 34% CaO, 4% P_2_O_5_, in mol.%) to achieve bioactivity and antimicrobial and antibacterial properties [138]. In a recent study [139], such PGLA fibers were also coated with composite coatings of Zn-doped BG and Ag-doped mesoporous BG-incorporated PCL or chitosan. The inclusion of ordered mesoporous BG particles can potentially allow for further loading of the system with drugs (e.g. anti-inflammatories or antibiotics) or growth factors, exploiting such mesoporous BG particles as drug carriers. Moreover, by implementation of the composite coating, the BG particles are stabilized on the fiber surface and the release of antibacterial ions can be properly tuned.

**Table 1:** BG reinforced PCL fibers for wound healing (studies reported after 2017)

**BG type**	**Polymer carrier**	**Improved biological properties**	**Reference**
45S5, Sr- and Mg-substituted BG (BGMS10), and Zn-substituted BG (BGMS-2Zn)	PCL	Improved cell adhesion and proliferation, higher wound healing rate	[131]
13-93B3	PCL	Improved cell (human adipose-derived mesenchymal stem cells) proliferation	[132]
B and Co co-doped bioactive glass nanoparticles	PCL	Upregulated VEGF and enhanced angiogenesis	[133]
77S	PCL	Improved cell (human skin fibroblast) adhesion and proliferation	[134]
Ag_2_O- and CoO-doped BG nanoparticles	PCL	Enhanced angiogenesis and antibacterial activity	[135]
45S5+ Cu nanoparticles	PCL	Improved cytocompatibility	[136]
58S	collagen/chitosan-coated PCL	Improved cell (human dermal fibroblast) proliferation and antibacterial activity	[137]

*BG* bioactive glass, *PCL* polycaprolactone, *VEGF* vascular endothelial growth factor

#### BG nano/microfibers

Cotton-wool-like BG fiber mats are an interesting type of inorganic fibers made of various BG compositions. They are typically made through a sol–gel process and by hydrolysis of alkoxide precursors, allowing bottom-up formation of a silicate glass network (gel) under ambient temperature [140]. After drying and calcination, nanoporous glasses in the form of a cotton-wool mat remain. Recently, such BG fiber scaffolds have been developed via electrospinning of a BG sol [141]. The BG composition could be formulated as (100-*x*)SiO_2_ – *x*CaO (*x* = 0, 10, 20, 30, and 40 mol.%). It was shown that the sol’s Ca content and relative humidity in the electrospinning chamber determine the morphology (and quality) of the cotton-wool-like fibers [141]. Taking 80S20C and 70SiO_2_-30CaO (70S30C) as two main BG compositions, the BG fibers co-cultured with HDF induced a similar cellular metabolic activity to that of the control sample (i.e. tissue culture polystyrene). Conversely, in the presence of such BG fibers, HDFs secreted a higher level of VEGF compared to the control [141].

In a very recent study, Ju et al. [142] synthesized antibacterial Ag-doped 70S30C BG fibers with a 3D cotton-wool-like structure through a combination of a sol–gel process and electrospinning. The as-prepared BG fibers can simulate the fibrous architecture of the skin’s ECM, and also enable moisture control and platelet aggregation in the wound bed [72,142]. Moreover, they release Ag and Ca/Si ions for antibacterial and wound healing purposes, respectively. Particularly, Ca ions upregulate fibrin and thrombin in the wound bed during the early stages of clot formation [143] and control the expression of various genes involved in epithelial migration [72]. Conversely, Si ions provoke proliferation of endothelial cells and upregulate the expression of VEGF and bFGF by fibroblasts, thereby enhancing angiogenesis [124,144]. In the field of silicate cotton-like fibers, recently, Cu-doped nanofibers were developed [96,107]. Other than silicate BGs, borate BGs, e.g. 13–93B3, have also been processed as cotton-candy-like fibers (however, not by electrospinning but via a glass melting process). Yang et al. [145] compared the *in vitro* behavior of 45S5 silicate BG fibers with that of 13-93B3 and 1605 borate BG fibers (the latter contains ZnO and CuO as dopants). According to this study, borate BG fibers were shown to release ions faster. Moreover, glass conversion and formation of HA in such fibers take place more promptly compared to silicate BG fibers. In general, borate BG fibers were proven to be more effective in terms of wound healing than silicate BG fibers. Such BG fibers have been shown to encourage the healing of full-thickness skin defects, thanks to the release of B and Ca ions that stimulate epidermal cell migration and angiogenesis, and govern the wound healing cascade [10]. B ions, in particular, drive the translation of encoding mRNA growth factors that trigger angiogenesis and wound healing including TGF-β and VEGF [146]. The BG nanofibers have been shown to offer an antibacterial activity, due to the increase of pH from 7 up to 9 and promotion of osmotic pressure of the tissue liquids by ionic dissolution [102]. Zhao et al. [41] developed Cu-doped borate BG microfibers (with the composition of 6Na_2_O, 8K_2_O, 8MgO, 22CaO, 54B_2_O_3_, 2P_2_O_5_; mol.%) that could release Cu, B and Ca ions into physiological medium, whereby enhancing the migration of HUVECs, tubule formation and secretion of VEGF. Moreover, these dissolution products could upregulate the expression of angiogenic genes in fibroblasts. Interestingly, it was shown that full thickness skin defects treated with such Cu-doped BG fibers achieved superior healing conditions reflected in larger collagen deposition, maturity and orientation. In addition to doping of the BG fibers (or particles) with pro-angiogenic elements, incorporation of angiogenic growth factors including VEGF, bFGF and PDGF into the tissue regenerating materials can be a second strategy to raise angiogenicity [54–56]. However, the implementation of growth factors imposes high costs and undesired biological consequences, e.g. in supra-physiological doses [57,58,147] and the incorporated factors might lose their bioactivity [59,60]. Therefore, with respect to BG-based fibers, doping of the BG particles with copper, for instance, can be regarded a superior strategy compared to incorporation of growth factors. Similarly, Ag-doped borate BG fibers (with the composition of 1–2 B_2_O_3_, 68–69 SiO_2_, ~0.001 Ag_2_O, and 29–30 CaO; mol.%) have been shown to offer an antibacterial activity and support the wound healing process [148].

Despite promising therapeutic effects of BG fibers, their mechanical mismatch with underlying skin tissue might cause an adverse effect on cellular behavior. As a proven fact, mechanical forces can control cell and tissue phenotype [149]. Cells employ an active contact sensing mechanism, whereby they respond to the stiffness of the underlying surface [150,151]. Specifically, dermal fibroblast cells respond to the substrate mechanics by altering their gene expression level, leading to differential ECM synthesis or their phenotypic transformation into myofibroblasts [152]. Moreover, migration of human bone marrow-derived mesenchymal stem cells, which accumulate and improve cutaneous wound healing [153], is governed by the mechanical properties of the ECM and depending on the matrix stiffness different differentiated phenotypes are generated [154]. Therefore, proper modulation of mechanical properties of a skin substitute or a dressing material can be vital in provision of an encouraging microenvironment for wound healing. One strategy to adjust the stiffness of the BG fiber dressings to match the underlying skin’s elastic properties could be hybridization of BG fibers with softer polymeric materials. This approach is not only efficient in terms of mechanical modulation, but also prevents uncontrolled release of ions from the BG fibers exposed to biological fluids. It has been shown that the Cu-doped borate BG fibers can release boron and Cu^2+^ ions in a non-tailored manner [155] and even trigger an initial burst release [156], thereby causing prompt degradation of the fibers and inducing transient biotoxicity [157]. Therefore, incorporation of BG fibers into a polymer matrix could be a more advantageous alternative for a BG fiber mat. In this regard, Hu et al. [158] developed vitamin E loaded Cu-doped borate BG microfibers incorporated poly(lactic-co-glycolic acid) wound dressings. According to *in vitro* tests, Cu^2+^ ions and vitamin were released in a sustained manner, thereby stimulating the secretion of VEGF in HUVECs and angiogenesis-linked genes in fibroblasts thus inducing better tubule formation. Moreover, the composite wound dressing was encouraging toward epithelialization and wound closure, collagen remodeling, and vessel sprouting *in vivo*.

### State-of-the-art and current challenges

BG (nano)fibers were originally investigated for the repair and restoration of hard tissues, mainly bone, thanks to their remarkable potential in formation of a surface HA layer. In recent years, BG fibers have also been proposed for development of soft tissue engineering scaffolds as well as wound dressings. It has been proven that the BG phase (depending on the composition) can release biologically active ions, thereby improving angiogenesis, which is an important prerequisite for wound healing. In this regard, bioresorbable borate-based BG fibers, commercially known as Mirragen®, have been successfully tested for chronic wound healing with commercial success. Mirragen® Advanced Wound Matrix (BBGFM) fabricated by ETS Wound Care (Rolla, Missouri) based on fibers (with composition: 53B_2_O_3_–6Na_2_O–12K_2_O–5MgO–20CaO–4P_2_O_5_ in wt.%) has been so designed to be degraded within a wound bed in days or weeks, depending on the wound exudate and healing rate [159].

Despite their superior bioactivity, BGs are predominantly fragile and suffer from low fracture toughness, particularly when formed as fibrous meshes. A promising solution can be the combination of BG particles with a supporting flexible polymeric phase. Synthetic and natural biodegradable polymers have been shown to perform properly as a carrier for BG (nano)particles and maintain ECM biomimicry. Moreover, hybridization of BG particles with a variety of polymers enables development of wound dressing materials with mechanical compatibility with natural skin tissue. Such dressing materials would be elastic, pliable and robust, thus protecting the wounded skin against mechanical damage. The mechanical properties of BG/polymer nanofibrous structures impact cellular activities thus tissue regeneration, because the cell–material interactions are largely dependent on the applied shear stresses and the mechanical signaling channels that govern the cells’ migration, proliferation and differentiation [85]. Therefore, mechanical properties of a wound dressing made from BG-based fibers must match the mechanical properties of the skin tissue to distribute comparable biomechanical signals [160]. As derived from the literature [161], human skin is as stiff as 0.1 to 10 MPa when exposed to tensile forces. Inorganic BG fibers are trivially much stiffer than skin but the BG-incorporated polymer (e.g. BG/PCL [137]) fibers can be so developed to properly match the skin’s elastic modulus. These properties can be adjusted in BG/polymer nanofibers to match that of the skin tissue through proper selection of polymer matrix, the quantity of the BG filler, and surface chemistry of the filler (in the presence/absence of coupling agents), which affects the physicochemical interaction (bonding) between the BG particles and the polymer. The fiber diameter could be also regarded as an influential factor, as it determines the arrangement of polymer chains in a confined area with a low free volume, thereby intensifying the entanglement of the polymer chains and their interaction with BG particles that would act as a physical barrier against mobility of the polymer chains.

Currently, doped (e.g. with Cu) and non-doped borate BG nanofibers are employed for wound healing applications. Depending on the specific healing objective, ranging from inflammation and proliferation to angiogenesis, there is a need for new customized formulations that can cover the entire wound healing process with a smart function triggered by exclusive environmental factors. For instance, pH and temperature can act as stimuli to induce the healing ion release, thereby governing the healing process in a controlled manner. In this regard, various ions can play a boosting role in each wound healing step including inflammation, proliferation and remodeling. Additionally, antibacterial ions can provide an extra functionality to BG-containing wound dressings as proposed also for sutures.

Regarding the metal ions doped into BG materials, it should be borne in mind that different metal ions perform only in a well-defined concentration range and if exceeded, the metal ion would engender undesired side effects. For instance, exceeding the optimum concentration range, Cu ions might generate free radicals, which are toxic to nerve cells, and raise the chance of neurodegenerative diseases [162]. Therefore, it is vital to notice the biosafety of metal ion-doped BG materials in the future, particularly given the complex nature of the human body’s internal physiological environment. In this regard, the design of BG-based fibers needs to be properly conducted to allow the release of ions in a controlled manner and within the safety and therapeutic relevant range.

Laser spinning and electrospinning are two main techniques for production of high-quality BG nanofibers. Electrospinning also enables production of hollow BG nanotubes and BG/polymer hybrid nanofibers. However, scalable production of BG nanofibers is still a challenge that potentially restricts their wide, commercial applications.

With respect to *in vivo* testing of BG nanofibers for wound healing, there is still a need of reliable animal models and novel skin on chip models. The currently used animal models such as mice, rats and rabbits do not realistically indicate the biological performance of the studied material in the human skin. Particularly, wound healing in mice is governed by myofibroblast-mediated contraction through an extensive subcutaneous striated muscle layer called the panniculus carnosus that is absent in humans [163]. Therefore, the biological results using this animal model cannot be extended directly to humans.

## Conclusions

Despite the well-known application potential of BG-based materials in hard tissue engineering, they are relatively new in the field of soft tissue repair and, in particular, as wound healing materials. In this context, BG fibers offer promising properties such as angiogenicity, immunogenicity and antibacterial activity, thus notably encouraging the skin repair cascade. This field is still in its infancy and further research in the future, particularly in relation to cotton-candy-like BG fibers, which are less studied compared to BG/polymer fibers, could pave the way toward the development of high-potential products for the dynamic wound dressing market. The successful studies reviewed in this paper indicate that bioactive glasses in the form of flexible (nano)fibers have a crucial role to play in the further progress of the field of antibacterial wound-healing biomaterials.

## Abbreviations

bFGF: Basic fibroblast growth factor; BG: Bioactive glass; ECM: Extracellular matrix; ESM: Eggshell membrane; HA: Hydroxyapatite; HCA: Hydroxycarbonate apatite; HDF: Human dermal fibroblasts; HIF: Hypoxia-inducible factor; HUVEC: Human umbilical vein endothelial cells; MC: Methylcellulose; PCL: Polycaprolactone; PDGF: Platelet-derived growth factor; PEO: Poly(ethylene oxide); PGLA: Poly(glycolide-l-lactide); PGS: Poly(glycerol sebacate); SBF: Simulated body fluid; VEGF: Vascular endothelial growth factor

## Data Availability

Not applicable.
